# Supplementary material for: The Effect of 24 h Shift Work on the Nutritional Status of Healthcare Workers: An Observational Follow-Up Study from Türkiye
Source: Nutrients. 2024 Jun 29;16(13):2088. doi: 10.3390/nu16132088 (PMC11243474; doi:10.3390/nu16132088)
Supplement: Supplementary file 1 [file nutrients-16-02088-s001.zip › nutrients-3065750-supplementary.pdf]

Supplementary Information

The effect of 24-hour shift work on the nutritional status of healthcare workers: an observational follow-up study from Türkiye

Semra Navruz Varlı[1,*]. Hande Mortaş[1]

[1]Gazi University, Faculty of Health Sciences, Department of Nutrition and Dietetics

*Corresponding author: semranavruz@gazi.edu.tr

Table S1. Distribution of healthcare professionals work areas according to the gender

Working area	Total (N:500) Number (%)	Female (n:361) Number (%)	Male (n:139) Number (%)
Emergency department	72 (14.4)	47 (13.0)	25 (18.0)
Ambulance	72 (14.4)	45 (12.5)	27 (19.4)
Surgery services	71 (14.2)	55 (15.2)	16 (11.5)
Internal services	123 (24.6)	94 (26.0)	29 (20.9)
Emergency call command control center	70 (14.0)	52 (14.4)	18 (12.9)
Intensive care	40 (8.0)	30 (8.3)	10 (7.2)
Palliative care	26 (5.2)	21 (5.8)	5 (3.6)
Units providing outpatient treatment (anesthesia, angiography, dialysis, others)	26 (5.2)	17 (4.7)	9 (6.5)

Table S2. Some information about working conditions, water consumption, and work stress of healthcare professionals according to gender

	Total (N:500)	Female (n:361)	Male (n:139)	
	Mean ± SD	Mean ± SD	Mean ± SD	p
Working time in the field (years)	6.3 ± 5.7	6.3 ± 5.8	6.3 ± 5.6	0.963
Number of days worked in shifts (number/month)	7.1 ± 2.5	7.2 ± 2.5	7.0 ± 2.6	0.655
Meal consumption amount on shift day (pieces/day)				
Main meal	2.4 ± 0.7	2.5 ± 0.7	2.5 ± 0.7	0.832
Snack	2.1 ± 1.1	2.2 ± 1.1	2.1 ± 1.1	0.267
Amount of water consumed (mL/day)	1644.7 ± 696.8	1546.1 ± 641.8	1900.7 ± 768.0	0.000*
Work stress score	24.7 ± 7.5	25.2 ± 7.4	23.3 ± 7.6	0.009*

*$p < 0.05$. SD: Standard deviation

Table S3. The most frequently preferred foods for main meals on shift and non-shift days

	Breakfast		Lunch		Dinner	
	Shift	Non-Shift	Shift	Non-Shift	Shift	Non-Shift
	Number (%)	Number (%)	Number (%)	Number (%)	Number (%)	Number (%)
Tea, cheese, olives, eggs, greens, etc.	257 (51.4)	405 (81.0)				
Tea, pastry, toast, bagel, etc.	250 (50.0)	180 (36.0)				
Only drink	51 (10.2)	38 (7.6)				
Cereal with milk	45 (9.0)	67 (13.4)				
Milk-yoghurt	34 (6.8)	21 (4.2)	151 (30.2)	155 (31.0)	160 (32.0)	190 (38.0)
Fruit	21 (4.2)	18 (3.6)	84 (16.8)	106 (21.2)	94 (18.8)	97 (19.4)
Soup, rice, pasta			333 (66.6)	313 (62.6)	309 (61.8)	320 (64.0)
Vegetables without meat			207 (41.4)	228 (45.6)	233 (46.6)	276 (55.2)
Vegetables with meat			218 (43.6)	231 (46.2)	253 (50.6)	297 (59.4)
Meat			208 (41.6)	209 (41.8)	234 (46.8)	264 (52.8)
Salads			238 (47.6)	236 (47.2)	247 (49.4)	289 (57.8)
Legume dishes			213 (42.6)	202 (40.4)	226 (45.2)	256 (51.2)
Fast food			79 (15.8)	66 (13.2)	166 (33.2)	140 (28.0)

Table S4. The most frequently preferred foods for snacks on shift and non-shift days

	Snack between breakfast and lunch		Snack between lunch and dinner		Night snack	
	Shift Number (%)	Non-Shift Number (%)	Shift Number (%)	Non-Shift Number (%)	Shift Number (%)	Non-Shift Number (%)
Candy, chocolate, wafers, etc.	145 (29.0)	117 (23.4)	141 (28.2)	134 (26.8)	127 (25.4)	104 (20.8)
Biscuits, crackers, chips, etc.	94 (18.8)	80 (16.0)	164 (32.8)	145 (29.0)	149 (29.8)	136 (27.2)
Toast, bagel, pastry, cheese in bread	83 (16.6)	84 (16.8)	61 (12.2)	43 (8.6)	84 (16.8)	63 (12.6)
Nuts	89 (17.8)	104 (20.8)	169 (33.8)	223 (44.6)	152 (30.4)	205 (41.0)
Fruit	84 (16.8)	81 (16.2)	161 (32.2)	215 (43.0)	182 (36.4)	246 (49.2)
Fruit juice, carbonated drinks	18 (3.6)	17 (3.4)	51 (10.2)	45 (9.0)	49 (9.8)	48 (9.6)
Milk, yoghurt, ayran	46 (9.2)	59 (11.8)	84 (16.8)	139 (27.8)	92 (18.4)	124 (24.8)
Tea, coffee	232 (46.4)	213 (42.6)	266 (53.2)	248 (49.6)	318 (63.6)	270 (54.0)

Table S5. Individuals' average daily consumption contents from food groups according to gender (g)

Food Groups	Female (n:361) Pre-shift Mean ± SD	Female (n:361) Shift Mean ± SD	Female (n:361) Post-shift Mean ± SD	Male (n:139) Pre-shift Mean ± SD	Male (n:139) Shift Mean ± SD	Male (n:139) Post-shift Mean ± SD
Meat and meat products. Eggs, legumes, and nuts/seeds	184.1 ± 120.5	218.3 ± 107.	144.6 ± 114.0	176.2 ± 100.9	218.5 ± 108.1	149.0 ± 105.2
Egg	44.7 ± 37.3	34.3 ± 31.5	30.6 ± 32.6	43.8 ± 36.1	35.8 ± 31.6	34.0 ± 30.2
Meat	69.4 ± 91.9	88.8 ± 70.7	57.4 ± 88.7	62.7 ± 63.7	95.2 ± 70.5	52.5 ± 63.6
Poultry	40.0 ± 78.0	61.6 ± 103.1	26.6 ± 64.0	41.6 ± 87.6	60.7 ± 104.6	33.3 ± 77.2
Fish	5.9 ± 32.9	3.6 ± 27.5	7.3 ± 40.2	5.5 ± 30.8	0.5 ± 6.7	9.6 ± 40.7
Legumes	13.6 ± 22.3	17.2 ± 30.7	11.6 ± 22.4	12.4 ± 21.3	13.3 ± 23.3	12.8 ± 23.2
Nuts/seeds	10.2 ± 18.5	12.5 ± 19.6	10.9 ± 23.7	10.1 ± 21.0	12.8 ± 20.5	6.5 ± 15.9
Milk and dairy products	209.6 ± 135.1	220.9 ± 148.0	183.7 ± 137.5	193.9 ± 118.2	245.5 ± 129.0	166.2 ± 129.2
Milk, kefir	58.5 ± 91.1	68.0 ± 97.7	63.8 ± 96.0	49.9 ± 84.3	57.5 ± 88.0	38.4 ± 68.5
Yogurt	116.0 ± 111.4	122.4 ± 117.3	97.0 ± 107.4	109.9 ± 101.2	152.4 ± 128.9	105.0 ± 110.8
Cheese	34.9 ± 31.6	30.5 ± 28.1	22.9 ± 25.8	34.1 ± 30.3	35.6 ± 30.9	22.7 ± 23.6
Bread and cereals	172.8 ± 92.7	165.4 ± 91.6	130.4 ± 79.0	183.0 ± 106.9	179.6 ± 90.8	134.7 ± 87.2
Bread	104.4 ± 74.3	88.1 ± 69.1	79.4 ± 64.3	99.1 ± 79.1	94.5 ± 67.3	77.1 ± 60.0
Bulgur	13.0 ± 27.0	15.9 ± 29.7	10.1 ± 22.2	14.2 ± 28.8	17.6 ± 31.3	6.0 ± 16.7
Rice	35.4 ± 54.9	39.8 ± 47.9	25.5 ± 43.0	48.1 ± 72.3	41.6 ± 56.5	35.1 ± 55.0
Pasta	19.9 ± 31.4	21.5 ± 35.5	15.3 ± 32.1	21.4 ± 34.0	25.8 ± 43.9	16.4 ± 35.8
Other	94.6 ± 50.0	91.2 ± 41.7	87.7 ± 41.2	129.8 ± 45.0	136.0 ± 64.2	88.5 ± 43.7
Vegetables	350.6 ± 206.1	376.9 ± 205.7	286.4 ± 189.0	392.7 ± 212.2	404.0 ± 202.9	303.4 ± 175.4
Fruits	122.0 ± 116.4	101.2 ± 98.4	106.6 ± 121.5	120.1 ± 115.9	92.6 ± 98.3	102.4 ± 118.5
Fats	47.4 ± 32.1	53.6 ± 34.0	41.6 ± 31.4	54.8 ± 32.1	53.5 ± 29.2	43.4 ± 34.0
Sugars	31.1 ± 33.2	41.0 ± 41.1	32.0 ± 34.7	36.4 ± 37.7	38.1 ± 41.5	23.0 ± 28.0

SD: Standard deviation
